# Computational Intelligence in Cancer Diagnostics: A Contemporary Review of Smart Phone Apps, Current Problems, and Future Research Potentials

**DOI:** 10.3390/diagnostics13091563

**Published:** 2023-04-27

**Authors:** Somit Jain, Dharmik Naicker, Ritu Raj, Vedanshu Patel, Yuh-Chung Hu, Kathiravan Srinivasan, Chun-Ping Jen

**Affiliations:** 1 School of Computer Science and Engineering, Vellore Institute of Technology, Vellore 632014, India; somit.jain2020@vitstudent.ac.in (S.J.); dharmik.naicker2020@vitstudent.ac.in (D.N.); ritu.raj2020@vitstudent.ac.in (R.R.); vedanshudaxesh.patel2020@vitstudent.ac.in (V.P.); kathiravan.srinivasan@vit.ac.in (K.S.); 2 Department of Mechanical and Electromechanical Engineering, National ILan University, Yilan 26047, Taiwan; ychu@niu.edu.tw; 3 School of Dentistry, College of Dental Medicine, Kaohsiung Medical University, Kaohsiung 80708, Taiwan; 4 Department of Mechanical Engineering and Advanced Institute of Manufacturing for High-Tech Innovations, National Chung Cheng University, Chia-Yi 62102, Taiwan

**Keywords:** deep learning, artificial intelligence, machine learning, computer-aided diagnostics, man-machine systems, cancer diagnosis, smart phone applications

## Abstract

Cancer is a dangerous and sometimes life-threatening disease that can have several negative consequences for the body, is a leading cause of mortality, and is becoming increasingly difficult to detect. Each form of cancer has its own set of traits, symptoms, and therapies, and early identification and management are important for a positive prognosis. Doctors utilize a variety of approaches to detect cancer, depending on the kind and location of the tumor. Imaging tests such as X-rays, Computed Tomography scans, Magnetic Resonance Imaging scans, and Positron Emission Tomography (PET) scans, which may provide precise pictures of the body’s interior structures to spot any abnormalities, are some of the tools that doctors use to diagnose cancer. This article evaluates computational-intelligence approaches and provides a means to impact future work by focusing on the relevance of machine learning and deep learning models such as K Nearest Neighbour (KNN), Support Vector Machine (SVM), Naïve Bayes, Decision Tree, Deep Neural Network, Deep Boltzmann machine, and so on. It evaluates information from 114 studies using Preferred Reporting Items for Systematic Reviews and Meta-Analyses extension for Scoping Reviews (PRISMA-ScR). This article explores the advantages and disadvantages of each model and provides an outline of how they are used in cancer diagnosis. In conclusion, artificial intelligence shows significant potential to enhance cancer imaging and diagnosis, despite the fact that there are a number of clinical issues that need to be addressed.

## 1. Introduction

According to the World Health Organization (WHO), as reported in Feb 2022, the most common cause of mortality in the world is cancer, causing nearly 10 million deaths in 2020: one in six deaths from all causes. By avoiding risk factors and putting into practice cancer prevention techniques that are already supported by research, it is already possible to prevent between 30 and 50 percent of cancer cases. The burden of cancer can also be reduced through the early detection of cancer as well as through the provision of proper treatments and care for people who have cancer. There are multiple studies on Deep Learning and Machine Learning techniques such as the Convolutional Neural Network (CNN) and Artificial Neural Network (ANN) based on early detection and prevention of cancer. Various types of cancer require different methodologies. For example, brain cancer can be detected through MRI images. In Appendix A, Table A1 presents a list of the acronyms used in this article along with their definitions.

### 1.1. Contribution of This Review

Our contribution can be summarized as follows:

1. We provide a comprehensive study on the various machine learning and deep learning models used for cancer diagnosis. A brief explanation of several machine learning and deep learning methodologies is included;
2. A contemporary review of cancer diagnosis as well as an application-oriented analysis of their datasets is given;
3. We highlight the current open challenges and future research possibilities in cancer diagnosis for aspiring researchers and enthusiasts to investigate;
4. We have covered almost all types of cancer diagnosis, which is a unique feature of our paper.

### 1.2. Review Methodology

Our contemporary review follows the PRISMA-ScR protocol, which is preferred for reporting items for systematic reviews and meta-analyses. It is a development of the original QUOROM guideline for contemporary reviews and meta-analyses of evaluations of healthcare solutions [1]. The PRISMA-ScR protocol includes items such as the study design, data sources, eligibility criteria, search strategies, data extraction and synthesis methods, risk of bias assessment, and the results and conclusions of the review. The protocol also includes guidance on the presentation of data, such as flow diagrams, and the reporting of funding and conflicts of interest.

**Figure 1:**
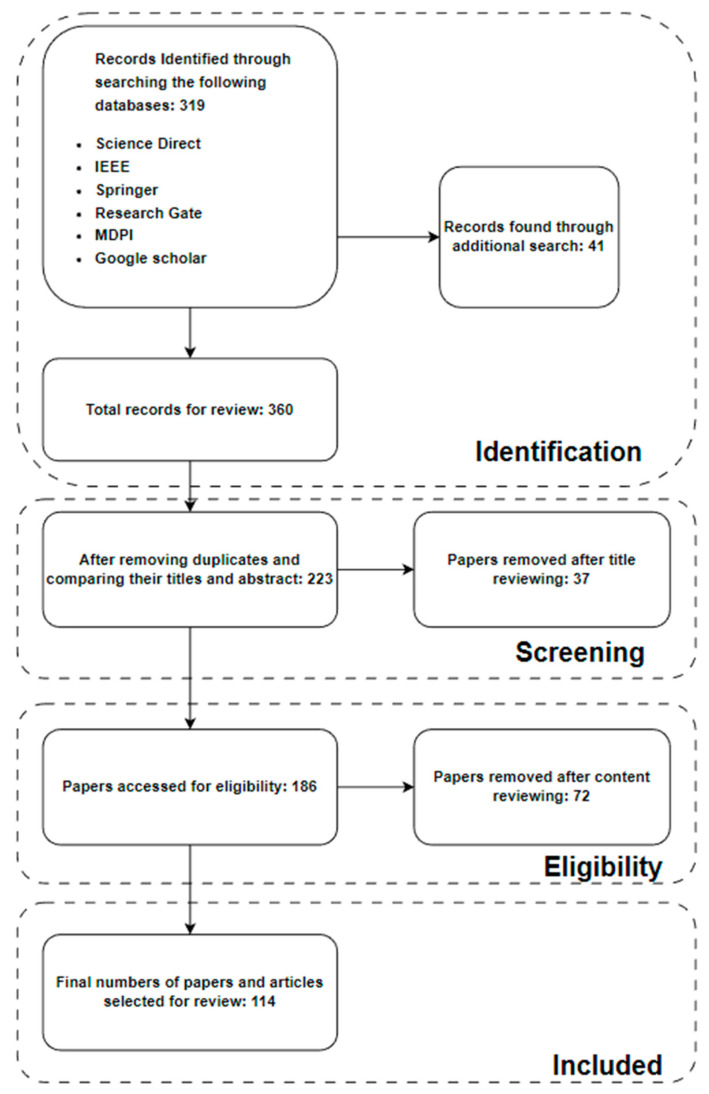
Flow diagram for the records identified by searching the database using PRISMA-ScR method.

PRISMA-ScR (Preferred Reporting Items for Systematic reviews and Meta-Analyses extension for Scoping Reviews) has a number of advantages over Joanna Briggs Institute (JBI) and other review methods. PRISMA-ScR offers a transparent and standardized reporting protocol to ensure that all pertinent information is reported in a plain and consistent manner. It is widely recognized and acknowledged by prominent organizations in the field of research synthesis, thereby enhancing the credibility and acceptability of scoping reviews. PRISMA-ScR also provides a comprehensive methodology for scoping reviews, including a clear definition of the research question, exhaustive search strategies, and systematic screening and data extraction procedures. These benefits make PRISMA-ScR a useful instrument for researchers conducting scoping reviews. Figure 1 shows the PRISMA-ScR method implementation.

### 1.3. Search Strategy and Literature Sources

For the purpose of finding pertinent publications, databases such as ScienceDirect, IEEE, Springer, Research Gate among others were explored. The keywords utilized were: Artificial intelligence, Computer-aided diagnostics, Deep Learning, Machine Learning, Man-machine systems, Cancer Diagnosis, and Smart Phone Apps. Initially, 360 unique articles were found from these databases.

#### 1.3.1. Inclusion Criteria

1. The significance of the articles determined their inclusion. The papers were chosen for this review depending on how novel they were;
2. Research comparing different ML, AI, and deep learning models for cancer diagnosis;
3. Relatively new articles were preferred;
4. English-language studies that were published in full text and reported accuracy and other performance measures.

#### 1.3.2. Elimination Criteria

1. The articles were eliminated because they were irrelevant;
2. Duplicate articles, non-English articles, or papers with sloppy writing;
3. No use of deep learning, machine learning, or AI models.

#### 1.3.3. Results

After deleting all duplicates, 223 papers remained from the 360 total papers retrieved (319 from databases and 41 from other searches). In addition, 37 articles were eliminated based on title screening, leaving 186 papers remaining. Around 72 papers were eliminated via content screening, which included the exclusion criteria, and 114 publications were ultimately utilized once all criteria were applied [1,2,3,4,5,6,7,8,9,10,11,12,13,14,15,16,17,18,19,20,21,22,23,24,25,26,27,28,29,30,31,32,33,34,35,36,37,38,39,40,41,42,43,44,45,46,47,48,49,50,51,52,53,54,55,56,57,58,59,60,61,62,63,64,65,66,67,68,69,70,71,72,73,74,75,76,77,78,79,80,81,82,83,84,85,86,87,88,89,90,91,92,93,94,95,96,97,98,99,100,101,102,103,104,105,106,107,108,109,110,111,112,113,114]. Figure 1 depicts the application of the same. The journal articles in Table 1 compare whether machine learning or deep learning models were utilized, marked by a Yes (✓) or No (×). It also includes the availability of open challenges and future directions in papers.

### 1.4. Structure of This Review

**Figure 2:**
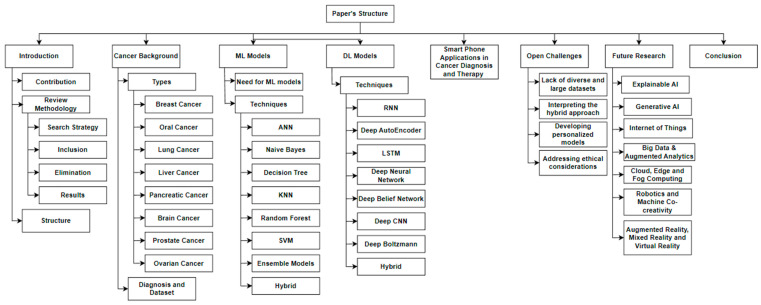
An illustration of the structure of this review.

Section 1 of this review discusses the introduction to cancer detection and the specifics of machine learning and deep learning, as well as open difficulties and future development. In addition, it explains how PRISMA-ScR is used to locate relevant publications. Section 2 describes several kinds of cancer and their phases. Comparative analyses of ML and DL approaches and their accuracies are presented in Section 3. Smart phone applications in cancer diagnosis and therapy are discussed in Section 4. Researchers can utilize Section 5 to obtain insight into the open difficulties in the field of cancer diagnosis. In Section 6, future research directions are discussed. Section 7 contains the conclusion, followed by the appendices and references. Figure 2 illustrates the structure of this review.

## 2. Cancer—Background

Cancer is a term used to describe a group of diseases characterized by the uncontrolled growth and spread of abnormal cells. The word “cancer” comes from the Latin word “carcinoma”, which means “a crab”. This term was used because cancer cells often resemble the legs of a crab, and cancer cells can spread and invade other parts of the body. 

There are many different types of cancer, and each type is classified based on the type of cell that is initially affected. For example, breast cancer begins in the cells of the breast, while lung cancer begins in the cells of the lung. Some other common types of cancer include prostate cancer, colon cancer, leukemia, and lymphoma. 

The exact cause of cancer is not fully understood, but it is believed to be the result of a combination of genetic mutations and environmental factors. Certain genetic mutations can increase a person’s risk of developing cancer, but these mutations do not always lead to cancer. Environmental factors, such as exposure to certain chemicals or substances, can also increase a person’s risk of developing cancer. Some viruses, such as human papillomavirus (HPV), can also cause cancer. 

Lifestyle factors also play a role in the development of cancer. For example, tobacco use is a major risk factor for several types of cancer, including lung cancer, throat cancer, and bladder cancer. Excessive alcohol consumption is also a risk factor for certain types of cancer, such as liver cancer and breast cancer. 

A healthy diet, regular physical activity, and maintaining a healthy weight can reduce the risk of developing cancer. In addition, undergoing regular cancer screenings, such as mammograms for breast cancer and colonoscopies for colon cancer, can help detect cancer early (when it is most treatable). 

The diagnosis of cancer typically involves a combination of medical imaging, biopsy, and laboratory tests. A biopsy is a procedure in which a sample of tissue is taken from a suspicious area and examined under a microscope to determine whether it is cancerous. Medical imaging, such as X-rays, CT scans, and MRIs, can help identify the location and size of a tumor. Laboratory tests can also be used to diagnose cancer, including blood tests and urine tests. 

Once a cancer diagnosis has been made, the next step is to determine the stage of the cancer. The stage of cancer refers to the size of the tumor and whether it has spread to other parts of the body. This information is important for determining the best treatment plan. 

Treatment for cancer often involves a combination of surgery, radiation therapy, and chemotherapy. Surgery is the removal of cancerous tissue and can be used for many types of cancer. Radiation therapy uses high-energy beams, such as X-rays, to kill cancer cells. Chemotherapy is the use of drugs to kill cancer cells. The choice of treatment will depend on the type and stage of the cancer, as well as the overall health of the patient. 

In some cases, targeted therapy or immunotherapy may also be used to treat cancer. Targeted therapy is a type of treatment that targets specific genes, proteins, or the tissue environment that contributes to the growth of cancer cells. Immunotherapy is a type of treatment that helps the body’s immune system fight cancer. 

Cancer can also have physical, emotional, and financial impacts on a person and their family. Physical side effects of cancer treatment, such as fatigue and nausea, can be managed with medications and other treatments. Emotional support, such as counselling and support groups, can also be helpful for people dealing with cancer. Financial support, such as financial assistance programs and insurance coverage, can also be important for people affected by cancer.

### 2.1. Cancer—Types

More than 100 different cancers exist. Typically, cancer types are named after the organs or tissues in which they first appear. For instance, brain cancer begins in the brain, and lung cancer begins in the lung. The type of cells that give rise to cancer, such as an epithelial cell or a squamous cell, can also be used to describe the condition.

#### 2.1.1. Breast Cancer

Breast tissue is where breast cancer typically develops in women and, less frequently, in men. A lump or thickening in the breast or underarm area; swelling, redness, or warmth in the breast; changes in the size or shape of the breast; dimpling or puckering of the breast skin; nipple change such as nipple inversion, discharge, or a rash around the nipple; and breast pain are common symptoms of breast cancer. 

There are normally five stages of breast cancer:

Stage 0: The cancer is non-invasive and confines itself to the breast tissue’s milk ducts or lobules;
Stage 1: refers to a cancer that is localized to the breast tissue, is tiny, and has not migrated to the lymph nodes or other regions of the body;
Stage 2: The cancer has not yet moved outside of the breast tissue, but it may have moved to adjacent lymph nodes;
Stage 3: Lymph nodes and more breast or chest-wall tissues have been affected by the malignancy;
Stage 4: The cancer has spread to the bones, lungs, or liver, among other organs.

#### 2.1.2. Oral Cancer

Oral cancer manifests in the tissues of the mouth or throat. It might have an impact on the cheeks, tongue, lips, gums, roof, or floor of the mouth. 

There are three phases of oral cancer:

Stage I: The cancer is minor, less than 2 cm in size, and limited to the site of genesis;
Stage II: The cancer is more advanced than stage I and often measures 2–4 cm;
Stage III: The cancer has grown to a size of 4 cm or has spread to adjacent lymph nodes;
Stage IV: The cancer has progressed to the liver or lungs, among other organs.

A sore or lump in the mouth that does not go away; discomfort when swallowing or chewing; white or red spots on the gums, tongue, or mouth lining; numbness in the mouth or face; persistent bad breath; loose teeth; and trouble wearing dentures are all indications of oral cancer.

#### 2.1.3. Lung Cancer

Lung cancer can manifest in two main forms: non-small cell lung cancer (NSCLC) and small cell lung cancer (SCLC). Symptoms and stages of lung cancer can differ based on the type and extent of the cancer, but typical signs of lung cancer consist of an ongoing cough, chest discomfort, shortness of breath, fatigue, unintentional weight loss, wheezing, and coughing up blood.

NSCLC can be staged in the following ways:

Stage 1: The cancer is only present in the lung and has not spread to other parts of the body or lymph nodes;
Stage 2: The cancer has disseminated to nearby tissues or lymph nodes within the chest;
Stage 3: The cancer has spread to organs in the chest or lymph nodes located in the middle of the chest;
Stage 4: The cancer has metastasized to other organs, including but not limited to the liver or bones.

On the other hand, SCLC has two stages:

Limited stage: The cancer is confined to only one lung, and several adjacent lymph nodes show malignancy;

Extensive stage: Distant organs such as the liver, brain, or bones have been affected by the cancer’s metastasis.

#### 2.1.4. Liver Cancer

The largest organ in the body, the liver, which is essential for filtering and processing blood, is where liver cancer typically develops. The types and stages of liver cancer can affect the signs and symptoms, but frequent signs and symptoms include stomach discomfort, swelling, nausea, vomiting, weakness, jaundice (a yellowing of the skin or eyes), itching, an enlarged liver, and a tumor in the abdomen.

Typically, liver cancer is divided into various stages:

Stage 1: The cancer is in its early stages and has not progressed beyond the liver;
Stage 2: The cancer may be more advanced than stage 1 but is still limited to the liver;
Stage 3: The cancer has metastasized to neighboring blood arteries or organs such as the pancreas or gallbladder;
Stage 4: The disease has spread to the bones, lungs, or brain, among other organs.

#### 2.1.5. Pancreatic Cancer

The pancreas, an organ behind the stomach that creates hormones such as insulin and glucagon as well as digestive enzymes, is where pancreatic cancer typically develops. Depending on the type and stage of the cancer, symptoms and stages can change. Back or stomach discomfort, loss of appetite or weight, nausea or vomiting, weakness or weariness, jaundice (yellowing of the skin or eyes), dark urine or light-colored feces, and newly established diabetes are all typical signs of pancreatic cancer.

Four phases are commonly used to categorize pancreatic cancer:

Stage 1: The pancreas is the only location where the cancer has spread;
Stage 2: The pancreas is still intact, but the cancer has migrated to neighboring lymph nodes;
Stage 3: The liver, spleen, stomach, or other surrounding blood vessels or organs have been affected by the malignancy;
Stage 4: The cancer has metastasized to additional tissues or organs, such as the lungs or bones.

#### 2.1.6. Brain Cancer

Brain cancer is one type of cancer that appears in the tissues of the brain or spinal cord. The symptoms and indications of brain cancer might change depending on the illness’s kind and stage. However, common signs and symptoms of brain cancer include headaches, which are frequently worse in the morning and get better with activity or after vomiting; nausea; vomiting; changes in vision, hearing, or speech; convulsions; seizures; weakness in the arms or legs; changes in personality or mood; and memory issues.

Based on the appearance of the cancer cells under a microscope and the rate of tumor growth, brain cancer is often divided into four grades:

Grade 1: The slow-growing cancer cells resemble healthy ones in appearance;
Grade 2: The cancer cells have a less-normal appearance and develop more quickly;
Grade 3: Cancer cells develop quickly and have a markedly different appearance compared to healthy ones;
Grade 4: The cancer cells show a high level of abnormality, are quickly growing, and spread.

#### 2.1.7. Prostate Cancer

The prostate gland, a little walnut-shaped organ in men that secretes seminal fluid, is where prostate cancer typically originates. It is a typical kind of cancer in men and might take years to develop slowly.

Prostate cancer has the following stages:

Stage I: The cancer is tiny and slow growing, localized to the prostate gland;
Stage II: The cancer has moved beyond the prostate gland but not to other bodily organs;
Stage III: The seminal vesicles or other surrounding tissues or organs have been affected by the malignancy;
Stage IV: The cancer has spread to other organs, including the lungs, liver, and bones.

Prostate cancer symptoms might include a difficult time urinating or a weak urine stream, blood in the urine or semen, pelvic or lower back pain, erectile dysfunction or trouble achieving or keeping an erection, and bone pain or fractures if the cancer has reached the bones.

#### 2.1.8. Ovarian Cancer

Ovarian cancer, a form of cancer that originates in the tissues of the ovaries, affects the ovaries, which are located on each side of the uterus and produce eggs and hormones. The signs and symptoms of ovarian cancer can vary depending on the type and stage of the disease, but common ones include abdominal or pelvic pain or swelling, bloating or a sudden feeling of fullness, changes in bowel or bladder habits, loss of appetite or weight loss, weakness or exhaustion, back pain, and menstrual changes.

There are normally four stages of ovarian cancer:

Stage 1: One or both ovaries are the only sites of the malignancy;
Stage 2: The cancer has metastasized to surrounding tissues such as the uterus or fallopian tubes;
Stage 3: The cancer has spread to the abdominal lining, surrounding lymph nodes, or further pelvic organs;
Stage 4: Distant organs such as the liver or lungs have been affected by the malignancy.

### 2.2. Cancer Diagnosis—Datasets

Table 2 shows the List of Various Cancer Diagnosis datasets.

## 3. Models for Deep Learning and Machine Learning in Cancer Diagnosis

**Figure 3:**
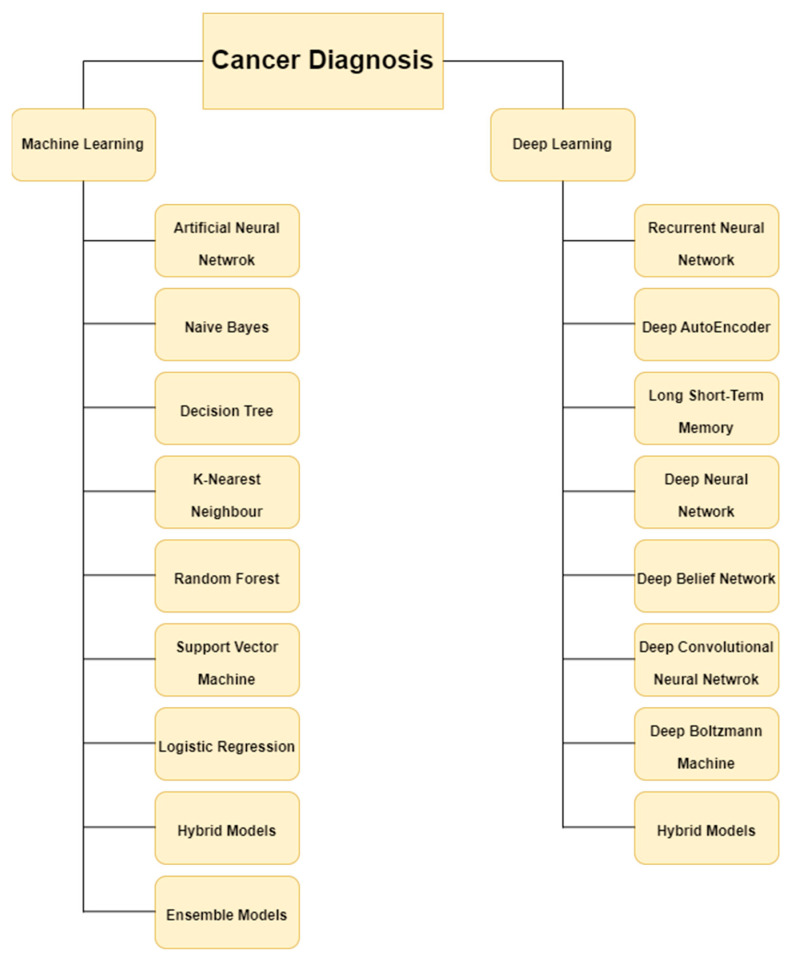
Machine learning and Deep Learning models used in various cancer diagnoses.

Figure 3 illustrates the machine learning and deep learning models used in various cancer diagnoses.

### 3.1. Requirement for Deep Learning and Machine Learning Models for Diagnosis of Cancer

The effectiveness and efficiency of cancer detection might be considerably enhanced with machine learning and deep learning models [8]. These models can evaluate vast volumes of data and find patterns and connections that human analysts cannot not instantly see. For instance, using machine learning and deep learning models, it is possible to examine medical pictures such as X-rays and CT scans and spot anomalies that could be cancerous growths. These models can also be trained on data from previous cancer cases to learn which characteristics are most indicative of a particular type of cancer. The accuracy of the diagnosis can be increased while saving time and costs by using deep learning and machine learning models for cancer diagnosis. This can ultimately lead to earlier detection and treatment of cancer, which can greatly improve patient outcomes [24]. 

### 3.2. Machine Learning Techniques

There are several machine learning techniques that can be used for cancer diagnosis. Some of these techniques include:

1. Supervised learning: This entails building a model using labelled data, where each input is paired with the appropriate output. For example, a model might be trained on a dataset of medical images that have been labelled as either normal or cancerous. The model can then be used to predict the likelihood of cancer for new images.
2. Unsupervised learning: This entails feeding a model unlabeled data and allowing it to determine linkages and patterns on its own. This can be useful for identifying unusual or unexpected patterns in medical data that may indicate the presence of cancer.
3. Reinforcement learning: This entails teaching a model to make decisions in a setting by rewarding or punishing specific actions. This can be used to optimize treatment plans for cancer patients based on their individual characteristics and needs.
4. Transfer learning: Instead of building a model from scratch, this method uses a model that has already been trained as the foundation for a new job. The pre-trained model can serve as a helpful basis for activities when there is a limited quantity of training data available.

Overall, machine learning techniques can be very powerful tools for improving the efficiency and accuracy of cancer diagnosis.

#### 3.2.1. Artificial Neural Network

ANNs can be trained to analyze medical images such as those from MRIs, CT scans, and X-rays to detect cancerous tumors. The system can learn to recognize patterns and characteristics in photos that are indicative of cancer by training ANNs on a huge dataset of medical images [25]. ANNs can be taught to assess genomic data to discover cancer-related mutations.

One advantage of using ANNs for cancer diagnosis is their ability to adapt and improve over time as more data become available. ANNs can also be trained on a variety of data types, making them a versatile tool for cancer diagnosis [26]. Table 3 illustrates the summary and accuracy of ANN model in cancer diagnosis.

#### 3.2.2. Naïve Bayes

Naïve Bayes is a probabilistic and classification algorithm that works by calculating the probability of a patient having cancer given their symptoms or test results [34].

One of the main advantages of using Naïve Bayes for cancer diagnosis is that it is a fast and efficient algorithm that can handle large amounts of data. It is also relatively simple to implement and can be used with a variety of data types.

**Figure 4:**
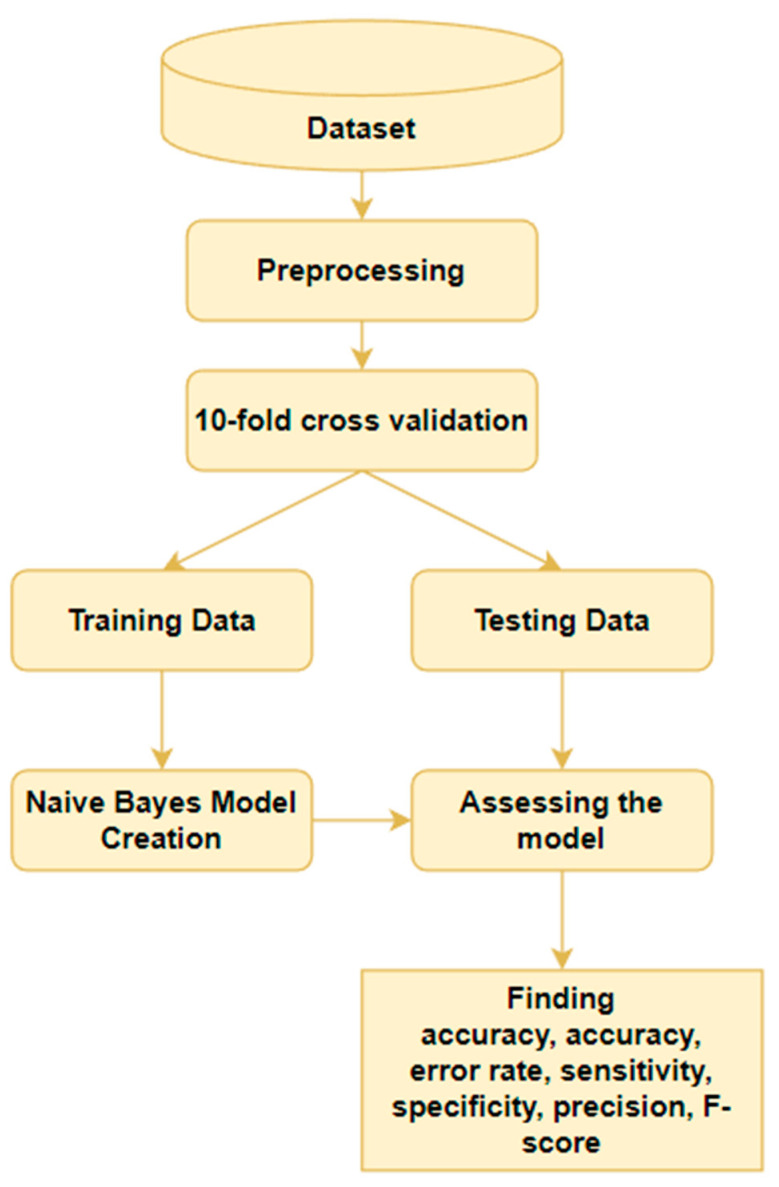
The progression of Naïve Bayes in cancer diagnosis.

However, it is important to note that Naïve Bayes has some limitations insofar as it assumes that all input features are independent of one another. This may not always be the case in medical data, and other machine learning algorithms may be more appropriate in some situations [35]. Overall, Naïve Bayes can be a useful tool for cancer diagnosis, particularly in cases when speed and efficiency are important. Table 4 illustrates the summary and accuracy of Naïve Bayes in cancer diagnosis and Figure 4 shows the progression of Naïve Bayes in cancer diagnosis.

#### 3.2.3. Decision Tree

Decision trees are very easy to interpret and can provide insight into which features are most important in making a diagnosis. This can be especially helpful for clinicians who need to make decisions about how to proceed with a patient’s care [39].

A decision tree works by dividing data into smaller and smaller groups based on the value of certain features or characteristics. For example, a decision tree might be used to predict the likelihood of breast cancer based on factors such as age, family history, and mammogram results. At each step in the tree, an algorithm makes decisions based on the data and splits the data into different groups accordingly [40]. The final prediction is made based on which groups the data fall into.

**Figure 5:**
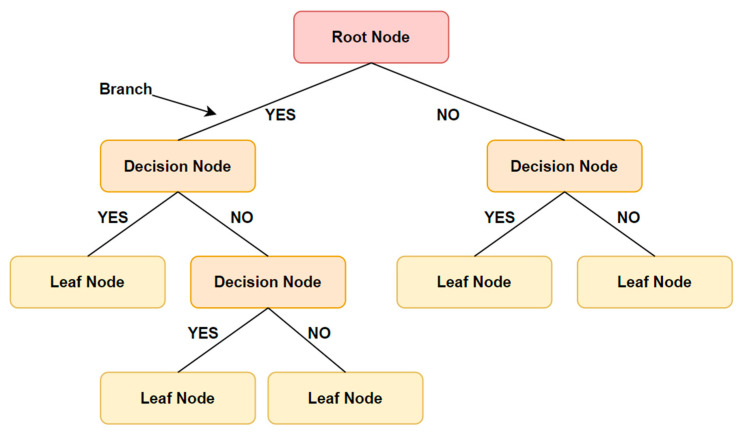
Decision Tree Model.

One of the main advantages of decision trees is that they are easy to understand and interpret, as they can be visualized in the form of a tree diagram. Moreover, decision trees are comparatively quick, effective, and able to process vast volumes of data. They might not generalize well to new data though, because they can be prone to overfitting. Overall, decision trees can be a useful tool for cancer diagnosis, particularly in cases when interpretability is important. Table 5 shows the summary and accuracy of cancer diagnosis using a decision tree. Figure 5 illustrates the decision-tree model.

#### 3.2.4. K-Nearest Neighbor

One potential advantage of KNN is that it can be useful for identifying rare subtypes of cancer or for identifying cases that are outliers or difficult to classify. This is because KNN uses similarity-based classification, wherein each new case is classified based on the similarities to its K nearest neighbors [44]. If a patient’s symptoms or test results are very different from those of other patients in the dataset, KNN may be better able to identify this patient as a unique case and provide a more accurate diagnosis.

One advantage of KNN is that it is simple to implement and can be used with a wide variety of data types. It is also relatively robust to noise and missing data. However, KNN can be computationally expensive, as it requires calculating distances between data points for each prediction [45]. It is also sensitive to the choice of K, which can impact the performance of the algorithm. Overall, KNN can be a useful tool for cancer diagnosis, particularly in cases when a simple and flexible algorithm is needed. Table 6 shows the summary and accuracy of cancer diagnosis using K-Nearest Neighbor. Table 6, row 1 explains how statistical feature extraction and linear kernels can be helpful in brain cancer detection using machine learning. However, when working with more complicated, non-linear data, Gabor wavelet feature extraction and RBF kernels can be more beneficial.

#### 3.2.5. Random Forest

Random Forest can handle many input features, even when some of those features may be weakly correlated with the outcome. This is important in cancer diagnosis, wherein there may be many factors that influence the likelihood of a patient having cancer when some of these factors may be difficult to measure or may have only a weak relationship with the outcome [48].

One advantage of Random Forest is that it is often accurate and resistant to overfitting. It is also fast and efficient and can handle large amounts of data. However, Random Forest models can be difficult to interpret as it is not always clear how the final prediction was made based on the individual decision trees. Overall, Random Forests are a powerful tool for cancer diagnosis and have been widely used in the field to improve the accuracy and efficiency of diagnosis. Table 7 shows the summary and accuracy of cancer diagnosis using Random Forest.

#### 3.2.6. Support Vector Machine

SVM can handle complex data distributions, including data that are not linearly separable. This is important in cancer diagnosis, wherein the relationship between the input features and the outcome can be highly complex and may not be easily captured by simpler algorithms.

SVM can handle multidimensional data and intricate relationships between the data points. Moreover, it is resistant to data noise and outliers. However, SVM can be computationally expensive and may not scale well to large datasets. Overall, SVM can be a helpful technique for detecting cancer, particularly in cases when the data have complex relationships and high-dimensional features [50]. Table 8 shows the summary and accuracy of cancer diagnosis using Support Vector Machine.

#### 3.2.7. Ensemble Models

Ensemble models can combine the predictions of multiple individual models, each of which may be specialized in a different way. This can decrease the danger of overfitting to the training data and increase the overall accuracy and robustness of the predictions. [56]. In cancer diagnosis, this can be particularly valuable in cases where the data are complex or noisy when a high degree of accuracy is required.

Ensemble models can be particularly useful in cancer diagnosis because they can improve the robustness and generalizability of the predictions. They can also be more accurate than individual models, as they can capture different patterns in the data and reduce the impact of noise or outliers. However, ensemble models can be more complex and may require more computational resources than individual models [57]. Overall, ensemble models can be a powerful tool for cancer diagnosis, particularly in cases when accuracy and reliability are important. Table 9 discusses the summary and accuracy of ensemble models for cancer diagnosis.

#### 3.2.8. Logistic Regression (LR)

Logistic regression (LR) is frequently used in cancer diagnostics to forecast the risk that a patient will develop a specific kind of cancer. Logistic regression has the benefit of allowing for the simultaneous analysis of several variables, which can aid in the identification of the most significant cancer predictors [59]. This can be especially helpful in the diagnosis of cancer, which may be affected by several risk factors, including age, family history, lifestyle, and environmental factors.

However, one drawback of utilizing logistic regression is that it may be constrained by the caliber and volume of accessible data [35]. The accuracy of logistic regression models’ future forecasts depends on the quality of the data used to train the model. The accuracy of the model may be hampered if the data are unreliable or skewed, and some patients’ cancer risks may not be correctly predicted. Moreover, logistic regression requires the assumption that the independent variables and the dependent variable have a linear relationship, which may not always be the case in real-world circumstances.

Although logistic regression has benefits for the diagnosis of cancer, such as the capacity to examine numerous variables at once, it is crucial to consider its drawbacks, particularly in relation to the caliber and volume of data needed to train the model. Instead of relying solely on the findings of a logistic regression analysis to make a diagnosis, healthcare providers should carefully consider the findings and utilize them as a tool to enhance clinical decision-making. Table 10 shows the summary and accuracy of cancer diagnosis using Logistic Regression.

#### 3.2.9. Hybrid Models

Hybrid machine learning (ML) models are being utilized more frequently in the diagnosis and treatment of cancer. Hybrid models, which combine the advantages of various ML models, can increase the precision of cancer diagnosis and aid in patient-outcome prediction. In contrast to conventional techniques, hybrid ML models have the benefit of processing and analyzing massive amounts of complicated medical data more quickly and precisely [60]. This can assist medical professionals in making more informed decisions on patient care, such as selecting the most efficient treatment strategy.

Hybrid ML models could have the drawback of being complicated and challenging to interpret. It can be difficult to comprehend how the model came to a specific diagnostic or prediction due to the integration of various models and algorithms [61]. This may influence patients’ ability to provide informed consent for treatments since it may be challenging for clinicians to communicate to patients the thinking behind their decisions. Additionally, some cancer forms may make it challenging to gather the vast quantities of high-quality data that hybrid ML models need to train and evaluate.

Overall, while utilizing hybrid ML models for cancer diagnosis may present some difficulties, the advantages of greater accuracy and quicker processing of medical data make them a promising option for enhancing cancer care. To give their patients the best care possible, medical professionals must carefully weigh the advantages and drawbacks of hybrid machine learning models and combine them with other diagnostic tools. Table 11 shows the summary and accuracy of cancer diagnosis using hybrid models.

Overall, depending on the unique data and situation, a variety of machine learning algorithms may be used. ANNs are a form of deep learning system that may be taught to recognize complicated data patterns. Based on medical information such as genetic markers and clinical test findings, Nave Bayes may be used to forecast the chance of a patient getting cancer. Decision trees are a classification and regression technique. KNN is a non-parametric method for classification and regression applications. KNN may be used in cancer diagnosis to estimate the risk of a patient having cancer based on similarities to other patients with confirmed cancer diagnoses. SVMs are algorithms that can perform classification and regression tasks.

In cancer diagnosis, machine learning algorithms may give useful insights and predictions, allowing physicians to make informed choices on how to proceed with a patient’s treatment.

### 3.3. Deep Learning Models

#### 3.3.1. Recurrent Neural Networks

In cancer diagnosis, these networks can analyze patient data and predict the probability of cancer based on certain characteristics or risk factors.

Ref. [63] mentions the use of fully connected, unrestricted designs and learning algorithms that can handle time-varying inputs and/or outputs in nontrivial ways using fully recurrent networks. Fully recurrent networks are nevertheless complicated for addressing complex issues, even though learning techniques have been modified to lower computational expenditure. Thus, partly recurrent networks, whose connections are primarily feedforward, with a carefully selected set of feedback connections, were utilized.

A Recurrent Neural Network (RNN) utilizes a recurrent connection to manage sequential data. The state vector is the RNN’s intermediate process. It assesses the combination of the values from the present input and the state vector from the past. The output is dependent on present input and previous input [63].

Overall, RNNs can be a useful model for the diagnosis of cancer, particularly in cases where there is a need to analyze sequential data and capture complex patterns in the data. However, it is important to note that RNNs are just one of many tools that can be used for cancer diagnosis, and the specific method or combination of methods used will depend on the specific context and needs. Table 12 shows the contribution and accuracy using recurrent neural networks for cancer diagnosis.

#### 3.3.2. Deep Autoencoder

One popular deep learning approach for feature learning is the deep autoencoder model. L1, L2, L3, L4, and L5 are the five layers that are intended to learn features from the original mass spectrum features. After preprocessing, the mass spectrum data serve as both the input and output vectors. The input dimensions of the OC 4302 and OC 8702 datasets are 9000 and 100,000, respectively. DAE and DCAE are the two different categories of autoencoder models. Whereas DCAE models have three convolutional layers, one max-pooling layer, and one up-sampling layer, DAE models have a 2000 × 1000 × 1000 structure [64].

The pooling size is set to 9, and the stride of the convolution operation is set to 1. The activation function for the PPGL dataset is chosen to be the ReLU function. Only 1000 nodes, or 1000 learnt features, make up the middle L3 layer, which is well-suited for classifying cancers. One advantage of using a deep autoencoder in cancer diagnosis is its ability to identify subtle patterns or anomalies in medical images that may not be visible to the human eye or may be difficult for radiologists to detect [65]. This can lead to earlier detection of cancer and more accurate diagnosis, which can improve patient outcomes and reduce the need for invasive procedures or treatments.

However, a disadvantage of using a deep autoencoder in cancer diagnosis is the potential for overfitting, which occurs when the model becomes too specialized to the training data and is unable to generalize to new data. This can result in false positives or false negatives, which can lead to incorrect diagnoses or missed diagnoses.

Overall, the use of a deep autoencoder for cancer diagnosis is a promising field of study that may enhance the precision and effectiveness of cancer detection. However, it is important to continue to refine and validate these models to ensure that they are reliable and effective in clinical practice [66]. Table 13 shows the summary and accuracy of cancer diagnosis using a deep autoencoder.

#### 3.3.3. Long Short-Term Memory

The detection of cancer is only one of the many uses for long short-term memory (LSTM) recurrent neural networks. One advantage of using LSTM in cancer diagnosis is its ability to analyze complex time-series data such as gene expression patterns or patient medical records. LSTM can process large volumes of data and recognize patterns in the data that may indicate the presence of cancer or other medical conditions [48]. This can potentially lead to earlier detection and more accurate diagnoses, which can improve patient outcomes.

One disadvantage of using LSTM in cancer diagnosis is that it can be computationally intensive and require significant computing resources. LSTM models can be complex and require significant amounts of training data to produce accurate results [69]. Additionally, LSTM models can be sensitive to overfitting, meaning that they may perform well on the training data but do not generalize well to new, unseen data. Therefore, it’s important to carefully design and train LSTM models to avoid these issues and ensure that they produce reliable and accurate results [70]. Overall, while LSTM has the potential to improve cancer diagnosis, careful consideration should be given to its design and implementation to ensure that it produces the desired results. Table 14 shows the summary and accuracy of cancer diagnosis using long short-term memory.

#### 3.3.4. Deep Neural Network

Deep neural networks (DNNs) are a type of machine learning model that can be trained to analyze complex datasets, including medical images and patient data. DNNs have shown promise in cancer diagnosis, particularly in the analysis of medical images such as mammograms and CT scans [70]. Since DNNs can pick up details in medical pictures that human eyes miss, they can be useful in the detection of cancer. This has the potential to increase the efficiency and precision of cancer diagnostics, ultimately benefiting both patients and doctors.

However, there are also some potential disadvantages to using DNNs in cancer diagnosis. One major concern is the potential for bias in the training data. If the DNN is trained on data that are not representative of the overall population, it may not be able to accurately diagnose cancer in certain patient groups [73]. Additionally, the complexity of DNNs can make it difficult to interpret how the model is making its diagnoses, which can make it challenging for healthcare professionals to understand and act on the results.

**Figure 6:**
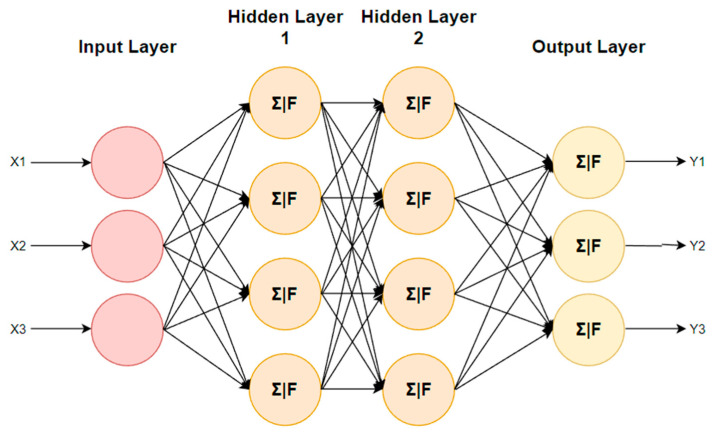
A deep neural network with multiple layers.

In conclusion, while there are both advantages and disadvantages to using DNNs in cancer diagnosis, ongoing research in this area holds promise for improving cancer detection and patient outcomes. It will be important for researchers and healthcare professionals to carefully consider the benefits and limitations of DNNs in the context of cancer diagnosis and to work to address any potential biases or other concerns that may arise. Table 15 shows the summary and accuracy of cancer diagnosis using deep neural networks. Figure 6 describes deep neural network with multiple layers.

#### 3.3.5. Deep Belief Network

To evaluate complicated data and generate predictions, scientists have developed artificial neural networks known as deep belief networks (DBNs). DBNs have been applied in a variety of fields, including cancer diagnosis, with both advantages and disadvantages.

One advantage to using DBNs in cancer diagnosis is their ability to analyze large amounts of data and identify patterns that may be missed by human experts [78]. For example, DBNs have been used to analyze gene expression data in cancer patients and predict the likelihood of disease recurrence or response to treatment. This can help doctors make more informed decisions about patient care and improve outcomes.

However, a disadvantage of using DBNs in cancer diagnosis is that they can be complex and difficult to interpret. DBNs rely on man-made parameters and hidden layers, and it can be challenging to understand how they arrive at their predictions [79]. This can make it difficult for doctors to explain their diagnoses to patients and for researchers to validate the accuracy of the models.

Overall, DBNs have shown promise in improving cancer diagnosis and treatment, but it is important to continue to develop and refine these models to ensure their accuracy and usefulness in clinical practice. Table 16 shows the summary and accuracy of cancer diagnosis using deep belief networks.

#### 3.3.6. Deep Convolutional Neural Network

In the fields of computer vision and image analysis, deep convolutional neural networks (DCNNs) are a popular form of artificial neural networks. In recent years, DCNNs have shown potential in analyzing medical pictures including mammograms, MRI scans, and pathology slides to aid the detection of cancer. One advantage of using DCNNs in cancer diagnosis is their ability to accurately detect subtle differences in images that may not be noticeable to the human eye [81,82]. This can help doctors and radiologists identify potential cancerous areas that might otherwise be missing, leading to earlier detection and more effective treatment.

However, there are also some potential disadvantages to using DCNNs in cancer diagnosis. One disadvantage is that the accuracy of the network is highly dependent on the quality and quantity of the training data used to develop the model. If the dataset is biased or limited in size, the network may not be able to accurately detect cancer or may make false-positive or false-negative diagnoses [83]. Additionally, the complexity of the network can make it difficult to interpret the results and understand how the network arrived at its diagnosis, which can be a concern for some clinicians and patients.

Despite these potential disadvantages, DCNNs have shown great potential in aiding cancer diagnosis and are continuing to be explored as a tool to improve cancer detection and treatment. Ongoing research is focused on refining the accuracy and interpretability of these networks, as well as exploring their potential use in other areas of medicine. Table 17 shows the summary and accuracy of cancer diagnosis using deep convolutional neural networks.

#### 3.3.7. Deep Boltzmann Machine

Deep Boltzmann Machines (DBMs) are a type of deep learning model that can be used in cancer diagnosis. One advantage of using DBMs in cancer diagnosis is that they can analyze large amounts of complex data, such as gene expression data, in a highly efficient manner. This can help to identify patterns or signatures that may be associated with specific types of cancer, and ultimately lead to more accurate and personalized diagnoses.

However, one disadvantage of using DBMs in cancer diagnosis is that they require a large amount of high-quality data to train the model effectively. This can be a challenge, as cancer data are often limited or of low quality, and collecting and processing data can be time-consuming and expensive [90]. Additionally, DBMs are highly complex models that require significant computational resources to train and run, which can be a barrier for smaller research institutions or clinics with limited resources.

Despite these challenges, the use of DBMs in cancer diagnosis holds promise for improving our understanding of cancer biology and developing more accurate and effective diagnostic tools. As with any new technology or approach, it is important to carefully evaluate the potential benefits and drawbacks of using DBMs in cancer diagnosis and to ensure that these methods are used ethically and responsibly [91]. Table 18 shows the summary and accuracy of cancer diagnosis using Deep Boltzmann Machines.

#### 3.3.8. Hybrid Models

Cancer diagnosis and treatment can be complex and time-consuming, requiring the expertise of multiple medical professionals and the use of various diagnostic tools. Hybrid deep learning models, which combine the strengths of different types of deep learning algorithms, have shown promise in improving the accuracy and efficiency of cancer diagnosis.

One advantage of using hybrid deep learning models in cancer diagnosis is that they can incorporate a wide range of data types and sources, such as medical images, electronic health records, and genomics data. By analyzing multiple types of data simultaneously, these models can provide a more comprehensive understanding of a patient’s cancer and its characteristics, allowing for more targeted and personalized treatment plans [68].

However, one disadvantage of using hybrid deep learning models in cancer diagnosis is that they can be computationally intensive and require significant resources, such as high-performance computing systems and large amounts of training data. Additionally, the complexity of these models can make them more difficult to interpret and explain, which may be a concern for medical professionals and patients who want to understand the reasoning behind a diagnosis.

Overall, hybrid deep learning models show great potential in advancing cancer diagnosis and treatment, but careful consideration of their advantages and disadvantages is necessary to ensure that they are used effectively and ethically in clinical settings. Table 19 shows the summary and accuracy of cancer diagnosis using hybrid deep learning models.

## 4. Smart Phone Applications in Cancer Diagnosis and Therapy

SkinVision is a mobile app that is available on iOS 13.0 or above and Android 7.0 or above. The app provides a partially free service that allows patients to track and analyze skin spots for potential signs of skin cancer [110]. Breast Check Now is a free mobile app that is available on iOS 10.0 or above and Android 4.4 or above. The app is designed to help women perform breast self-exams and raise awareness about breast cancer [111]. Ada Health is a free mobile app that is available on iOS 13.0 or above and Android 5.0 or above. The app serves as a symptom assessment tool and a health library, providing patients with a comprehensive resource for assessing their symptoms and understanding potential health issues [112].

Cancer.Net Mobile is a free mobile app that is available on iOS 10.0 or above and Android 5.0 or above. The app provides cancer-related resources and tools for tracking symptoms, appointments, and medications. It is designed for use by both patients and physicians, making it a valuable resource for anyone affected by cancer [113]. ASCO Guidelines is a free mobile app that is available on iOS 12.0 or above and Android 5.0 or above. The app provides physicians with tools for risk assessment, staging, and treatment-related decision-making based on the latest clinical evidence [114]. Table 20 shows the various smart phone applications available.

## 5. Open Challenges—Computational-Intelligence-Based Cancer Diagnosis

There is also the issue of who should be held accountable (the doctor or the computer) in the event of an inaccurate AI-based diagnosis. The adoption of AI into oncology practice also impacts patients’ rights to make their own healthcare decisions and their relationships with their clinicians. Another challenge involves relevant datasets that have adequate amount of data for processing into various ML and deep learning models.

**Figure 7:**
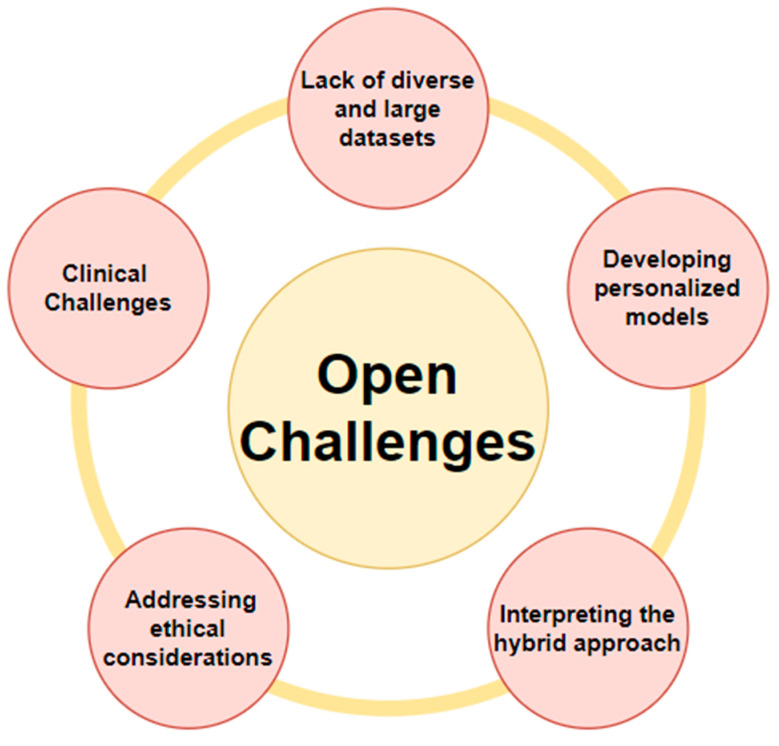
Open challenges in the diagnosis of various cancers.

Further image processing still must be performed more precisely for better classification results for new data. Figure 7 illustrates the open challenges in cancer diagnosis.

### 5.1. Lack of Diverse and Large Datasets

According to [97], in order to be effectively trained, deep learning algorithms require datasets that are both large and diverse. On the other hand, there are only a limited number of such datasets available, which can make it more difficult to develop accurate deep learning models for the detection and diagnosis of skin cancer. The development of datasets that are both larger in scope and more varied in composition could be one solution to this problem.

### 5.2. Interpreting the Hybrid Approach

Similar to many other approaches, the hybrid approach in [98] proposed by Sahu and colleagues brings together machine learning algorithms and the knowledge of subject-matter experts. This strategy has the potential to be successful; however, the reasons why the algorithm made its decision are not always obvious. Creating methods for interpreting the hybrid approach is one way to help increase trust in the decisions that are produced by the algorithm.

### 5.3. Developing Personalized Models

As can be clearly interpreted from [99], mammographic breast-cancer-diagnosis models frequently assume that patients’ presenting characteristics are distributed in the same way. However, different patients might have one-of-a-kind characteristics that are not taken into account by the model. It may be possible to improve the accuracy of mammographic breast cancer diagnosis by developing new methods for the creation of personalized models that can take into account the unique characteristics of each individual patient.

### 5.4. Addressing Ethical Considerations

Patient privacy issues are ethical. Machine learning algorithms require medical history, imaging, and genetics. Unauthorized access or security breaches might harm patients. Software developers must limit data breaches and anonymize patient data to remedy this. Patients should also know how their data will be used and opt out. Another ethical challenge in cancer-detection-machine-learning systems is bias. If the training data are not representative of the patient population or do not account for demographic or socioeconomic characteristics that may impact cancer detection, the algorithm may be biased. Software engineers must train their algorithms on patient population data sets to reduce this danger. Simple algorithms assist patients and professionals in comprehending diagnoses and data analyses. Misdiagnosis by an algorithm, e.g., in brain tumors, can make defect identification difficult [109]. Algorithm developers must explain their results and accept accountability. Doctors must understand diagnostic algorithms and be accountable.

### 5.5. Clinical Challenges

AI models trained on one patient population may not work well on another. In cancer imaging, wherein disease presentation and treatment responses vary widely, this might be difficult. Regarding the interpretability of AI models, complex AI models might make it difficult for physicians to grasp how they reached a diagnosis or therapy suggestion. This can also make model output difficult to trust and incorporate into clinical decision making. AI models must be integrated into clinical processes efficiently and without disrupting current practices. This may involve adding imaging tests or adjusting test schedules, which might be difficult to accomplish. Privacy, data security, and liability issues arise when using AI in clinical practice. AI model regulatory approval and clinical usage may be difficult [106].

## 6. Future Research Directions

### 6.1. Explainable AI

There are several potential directions for future research in the use of explainable artificial intelligence (AI) for cancer diagnosis:

1. Developing more interpretable models: One goal of explainable AI is to create models that are more transparent and interpretable so that the decision-making process can be better understood by humans. In the field of cancer diagnosis, this could involve developing models that are more easily explainable, such as decision tree-based models or linear models;
2. Improving transparency and accountability: Another goal of explainable AI is to improve the transparency and accountability of AI systems. In the context of cancer diagnosis, this could involve developing methods for explaining the predictions made by AI systems in a way that is understandable to both doctors and patients;
3. Integrating human expert knowledge: Another direction for future research could involve integrating human expert knowledge into explainable AI systems. For example, this could involve developing models that can incorporate expert knowledge about cancer diagnosis into their decision-making processes in order to improve the accuracy and reliability of the predictions;
4. Improving the efficiency of explainable AI: Finally, there is also a need to improve the efficiency of explainable AI systems, so that they can be used in practical applications. This could involve developing methods for reducing the computational cost of explainable AI models or developing methods for scaling these models to larger datasets [100].

### 6.2. Generative AI

There are several potential directions for future research in the use of generative artificial intelligence (AI) for cancer diagnosis:

1. Improving the accuracy of generative models: One goal of generative AI is to create models that can accurately generate realistic data, such as images or text. In the field of cancer diagnosis, this could involve developing generative models that are able to generate high-quality medical images or other types of patient data that are useful for diagnosis [101];
2. Developing more efficient generative models: Another direction for future research could involve developing more efficient generative models, which can generate data more quickly or with fewer computational resources. This could be particularly important in the context of cancer diagnosis, wherein time is often of the essence and there is a need to process large amounts of data quickly;
3. Integrating generative models with other AI approaches: Another potential direction for future research could involve integrating generative models with other types of AI algorithms, such as discriminative models or reinforcement-learning algorithms. This could allow for the development of more powerful and flexible AI systems for cancer diagnosis;
4. Applying generative models to new types of data: Finally, there may also be opportunities to apply generative models to new types of data that have not been used extensively in the past for cancer diagnosis [102]. For example, this could include data from wearable sensors, social media, or other sources that have not been widely used in the past.

### 6.3. Internet of Everything

There are several potential directions for future research in the use of the Internet of Everything (IoE) for cancer diagnosis:

1. Improving data security and privacy: One of the main challenges in using the IoE for cancer diagnosis is ensuring the security and privacy of patient data. This could involve developing methods for encrypting and protecting data as they are transmitted over the internet as well as developing protocols for controlling access to sensitive data;
2. Developing new sensors and devices: Another direction for future research could involve developing new types of sensors and devices that can be used to collect data for cancer diagnosis. This could include wearable sensors, such as smart watches or fitness trackers, as well as more traditional medical devices, such as imaging machines or laboratory equipment;
3. Integrating data from multiple sources: Another potential direction for future research could involve developing methods for integrating data from multiple sources, including both traditional medical sources and non-medical sources, such as social media or wearable devices. This could allow for a more comprehensive and accurate view of a patient’s health status;
4. Improving the interoperability of devices: Finally, there is also a need to improve the interoperability of devices and systems within the IoE so that they can communicate and exchange data more easily. This could involve developing standards and protocols for data exchange, as well as developing methods for enabling devices from different manufacturers to work together seamlessly [103].

### 6.4. Big Data and Augmented Analytics

There are several potential directions for future research in the use of big data and augmented analytics for cancer diagnosis:

1. Improving data quality and accuracy: One challenge in using big data for cancer diagnosis is ensuring the quality and accuracy of the data. This could involve developing methods for cleaning and pre-processing data, as well as developing methods for verifying the accuracy of the data;
2. Developing personalized treatment plans: One potential application of big data and augmented analytics in cancer diagnosis could be the development of personalized treatment plans based on a patient’s unique characteristics and risk factors. This could involve analyzing large amounts of data to identify patterns and trends that are specific to individual patients and using this information to tailor treatment recommendations;
3. Improving the accuracy of prognoses: Another potential use of big data and augmented analytics could be to improve the accuracy of prognoses for cancer patients. This could involve analyzing large amounts of data to identify factors that are associated with better or worse outcomes and using this information to make more accurate predictions about a patient’s likely course of treatment and outcome;
4. Identifying new treatment options: Big data and augmented analytics could also be used to identify new treatment options for cancer patients. For example, by analyzing large amounts of data from clinical trials and other sources, it may be possible to identify promising new treatments that have not yet been widely adopted;
5. Developing new drug compounds: Finally, big data and augmented analytics could be used to develop new drug compounds for the treatment of cancer. This could involve analyzing large amounts of data on the molecular mechanisms of cancer, as well as data on the effects of different drug compounds, to identify potential candidates for further development [104].

### 6.5. Cloud, Edge, and Fog Computing

There are several potential directions for future research in the use of cloud, edge, and fog computing for cancer diagnosis:

1. Improving data security and privacy: One challenge in using cloud, edge, and fog computing for cancer diagnosis is ensuring the security and privacy of patient data. This could involve developing methods for encrypting and protecting data as they are transmitted between different devices and servers as well as developing protocols for controlling access to sensitive data;
2. Improving data interoperability: One potential direction for future research could involve developing methods for improving the interoperability of data between different devices and servers in cloud, edge, and fog computing environments. This could involve developing standards and protocols for data exchange as well as developing methods for enabling devices from different manufacturers to work together seamlessly;
3. Enabling real-time data analysis: Another potential application of cloud, edge, and fog computing in cancer diagnosis could include enabling real-time data analysis. This could involve using these technologies to process and analyze data as they are being collected in order to make more timely and accurate diagnoses;
4. Developing new types of distributed systems: Another direction for future research could involve the development of new types of distributed systems that are specifically designed for use in healthcare applications such as cancer diagnosis. This could include the development of new types of cloud, edge, and fog computing architectures as well as new types of software and hardware tools for these systems;
5. Enhancing the scalability of distributed systems: Finally, there may also be opportunities to enhance the scalability of distributed systems in cloud, edge, and fog computing environments. This could involve developing methods for scaling these systems to larger datasets or more complex workloads in order to support the increasing demands of healthcare applications [105].

### 6.6. Robots and Machine Co-Creativity

There are several potential directions for future research in the use of robots and machine co-creativity for cancer, for example:

1. Developing new co-creativity algorithms: Another potential direction for future research could involve the development of new algorithms and software tools that enable robots and humans to work together more effectively in the context of cancer diagnosis and other healthcare applications. This could involve the development of machine learning algorithms or other types of artificial intelligence (AI) tools that are specifically designed for co-creative tasks;
2. Enhancing the safety and reliability of robots: There is also a need to focus on the safety and reliability of robots in healthcare applications in order to ensure that they can be used safely and effectively. This could involve developing methods for testing and verifying the performance of robots as well as developing methods for mitigating potential risks and hazards;
3. Enhancing the flexibility and adaptability of robots: Another potential direction for research could involve developing methods for improving the flexibility and adaptability of robots in healthcare applications. This could involve the development of new algorithms and software tools that enable robots to adapt to changing environments or tasks as well as new types of sensors and actuators that allow for greater flexibility and adaptability;
4. Developing new ways to incorporate human expertise: Another potential direction for future research could involve developing new ways to incorporate human expertise in the decision-making process of robots in healthcare applications. This could involve the development of new machine learning algorithms or other types of AI tools that are able to learn from human experts or the development of new types of user interfaces that enable humans to collaborate more effectively with robots;
5. Enhancing the reliability and maintainability of robots: Finally, there is also a need to focus on the reliability and maintainability of robots in healthcare applications. This could involve developing methods for testing and verifying the performance of robots as well as developing methods for maintaining and repairing these systems when necessary.

### 6.7. Augmented Reality (AR), Mixed Reality (MR), and Virtual Reality (VR)

There are several potential directions for future research in the use of augmented reality (AR), mixed reality (MR), and virtual reality (VR) for cancer diagnosis. Some possible areas of focus could include the following:

1. Enhancing the capabilities of existing AR, MR, and VR systems: Another potential direction for research could involve enhancing the capabilities of existing AR, MR, and VR systems by improving their accuracy, resolution, or field of view. This could involve the development of new software and hardware tools as well as new types of sensors and displays;
2. Developing new interaction techniques: One potential direction for future research could involve developing new interaction techniques that enable users to more effectively interact with AR, MR, and VR systems in healthcare applications. This could involve the development of new types of gesture recognition or voice control algorithms as well as new types of haptic interfaces;
3. Enhancing the realism of virtual environments: Another potential direction for research could involve developing methods for enhancing the realism of virtual environments in AR, MR, and VR systems. This could involve the development of new graphics and rendering algorithms as well as new types of sensors and displays that enable more realistic representations of physical objects and environments;
4. Enhancing the usability and user experience of AR, MR, and VR systems: Finally, there is also a need to focus on the usability and user experience of AR, MR, and VR systems in healthcare applications. This could involve developing methods for improving the ease of use and comfort of these systems, as well as developing methods for enhancing the realism and immersion of virtual environments.

**Figure 8:**
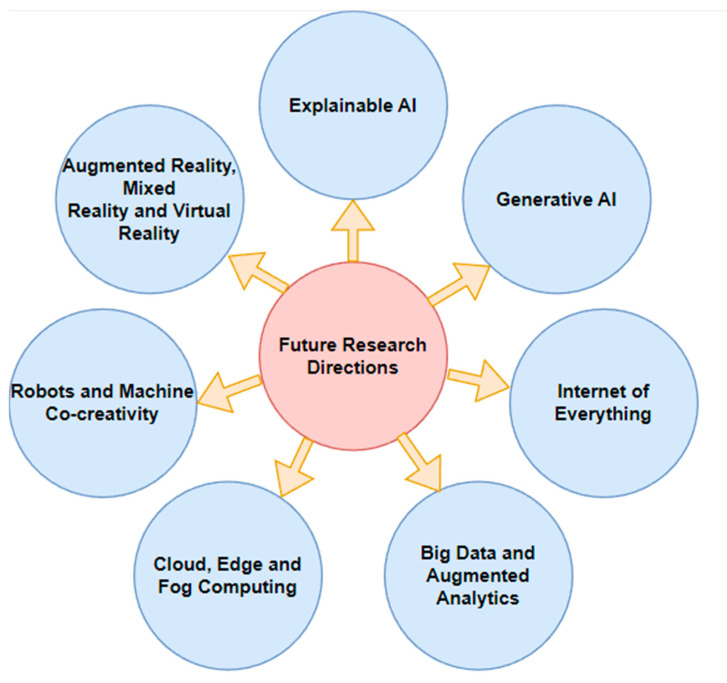
Future research directions for the diagnosis of various cancers.

Figure 8 illustrates the various future research directions in the field of cancer diagnosis.

## 7. Conclusions

In conclusion, the use of machine learning and deep learning models for cancer diagnosis has shown promising results in recent years. The results of this contemporary review indicate that these models can accurately diagnose various types of cancer, including breast cancer, lung cancer, and oral cancer. However, it is important to note that these models are still in the early stages of development, and further research is needed to validate their accuracy and improve their performance. Despite this, the potential benefits of using machine learning and deep learning models for cancer diagnosis, such as increased accuracy and improved efficiency, make them a promising area of study. We observed that machine learning and deep learning models achieved an average accuracy of 90.01% and 90.46%, and the machine learning model with best average accuracy was Naïve Bayes. Further research should be conducted to continue to evaluate the performance of these models and to identify potential barriers to their implementation in clinical practice and also on the hybrid models that not only use a single model but multiple models to increase accuracy.

## Figures and Tables

**Table 1:** Comparison with earlier studies on the application of AI in cancer diagnosis.

Reference	Year	One-Phrase Summary	Machine Learning	Deep Learning	Open Challenges	Future Directions
Our Review	-	This review explores the advantages and disadvantages of each model and provides an outline of how they are used in cancer diagnosis. It finishes with future work on the topic, outstanding questions, and potential for further study.	**✓**	**✓**	**✓**	**✓**
[2]	2021	This review highlights the use of a variety of imaging techniques and data analysis methods as it investigates the potential of radiomics and machine learning for investigating radiation-induced toxicity in head- and neck-cancer patients. According to the review, radiomics and machine learning have the potential to help improve our understanding of radiation-induced toxicity and assist in guiding treatment decisions in patients such as these.	**✓**	**×**	**×**	**×**
[3]	2021	The primary focus of this investigation is on visual data, and the CNN algorithm is currently the most widely used method for categorizing visual data.	**✓**	**✓**	**×**	**×**
[4]	2021	This paper provides a comprehensive and methodical review of deep learning approaches that can aid in the early detection of skin cancer.	**×**	**✓**	**✓**	**✓**
[5]	2021	This paper employs two approaches. The first approach uses radiobiological models such as those for tumor control probability (TCP), normal tissue complication probability (NTCP), and equivalent uniform dose. The second approach constructs statistical models that make use of all the information that is pertinent to disease prognosis, such as demographic information, laboratory test results, image data, and dosimetry, in order to determine the relationship between these factors and clinical outcomes.	**✓**	**×**	**✓**	**✓**
[6]	2021	This review includes topics related to the anatomy of brain tumors, including publicly accessible datasets, augmentation techniques, segmentation, feature extraction, classification, deep learning, transfer learning, and quantum machine learning for analysis.	**✓**	**✓**	**✓**	**✓**
[7]	2021	In this paper, emphasis is placed on presenting potentially game-changing technology of the future in clinical settings by bridging the gap between biology and artificial intelligence and explaining how AI-based assistance can assist oncologists in providing precise therapy.	**✓**	**×**	**✓**	**✓**
[8]	2020	Using machine learning, including supervised, unsupervised, and deep learning approaches, the process of diagnosing and curing cancer is facilitated in this paper.	**✓**	**✓**	**✓**	**×**
[9]	2020	This paper emphasizes Raman Spectroscopy and ML for better medical diagnostics in cancer.	**✓**	**×**	**✓**	**✓**
[10]	2020	This paper uses GoogleNet from CNN to train cancer images in a type of learning called “transfer learning.” A cancer-imaging database is used to pull out multimodal features such as texture, morphology, entropy, SIFT, and EFDs. The performance of both single features and combinations of features are evaluated using strong machine learning techniques. SVM Gaussian is used, along with a number of different features, including texture, morphological, and EFDs, in order to perform the machine learning analysis that gives the best results, with a sensitivity and total accuracy of 99.71%, followed by SVM Gaussian with texture + SIFT features, which gives a sensitivity of 98.83% and an area under the curve (AUC) of 0.999. When GoogleNet is used with the Deep Learning CNN approach, the results are 100% specificity, sensitivity, PPV, and TA and an AUC of 1.00.	**×**	**✓**	**✓**	**✓**
[11]	2019	This paper attempts to outline the pathophysiology, imaging modalities, and automatic computer-assisted approaches for the characterization of brain cancer within the context of machine learning and deep learning.	**✓**	**×**	**✓**	**✓**
[12]	2018	According to the findings of this paper, analysis using convolutional neural networks (CNN) has the greatest number of applications in deep learning and medical imaging.	**×**	**✓**	**×**	**×**
[13]	2017	In this paper, ML is utilized to aid in the surgical treatment of epilepsy, brain tumors, spinal lesions, neurovascular pathology, Parkinson’s disease, traumatic brain injury, and hydrocephalus.	**✓**	**×**	**✓**	**✓**

**Table 2:** List of Various Cancer Diagnosis datasets.

References	Dataset	Type of Dataset	Key Points Regarding the Dataset
[14]	TCIA	Radiomic	TCIA consists of a collection of medical images, which includes CT scans of people with lung cancer. The dataset contains data on the characteristics, diagnosis, and course of treatment for patients.
[15]	NLST	Radiologic and clinical data	The National Lung Screening experiment (NLST) dataset is a large-scale clinical experiment that was conducted at multiple institutions to examine the efficacy of low-dose CT screening for lung cancer. The collection contains CT scans obtained from approximately 53,000 participants, which include both those who had cancer and healthy controls.
[16]	LUNA16	Radiomic	LUNA16 contains 888 CT scans, each with a resolution of 512 × 512 pixels and a slice thickness of 0.5–3.0 mm. The dataset includes annotations for nodules that indicate information such as where the nodule is, how big it is, and how likely it is to be cancerous.
[7]	ChEMBL and PubChem	Clinical and Genomic	Both ChEMBL and PubChem are large databases with significant amounts of information concerning chemical compounds and their functions in living things. It contains carefully chosen information about the properties and actions of small molecules such as drugs and drug candidates, natural products, and man-made compounds. Bioassay data, including how active a compound is against a certain target or in a certain cell line, are also included in ChEMBL.
[17,18]	BRATS 2015, 2016	Radiomic	BRATS 2015 and 2016 contain 220 high-grade and 54 low-grade glioma MRIs.
[19]	BRATS 2017	Radiomic	BRATS 2017 consists of 285 brain-tumor MRI scans.
[20]	BRATS 2018	Radiomic	With each case including four MRI modalities, the BRATS 2018 dataset consists of 3D brain MRIs and ground-truth brain tumor segmentations annotated by physicians.
[21]	LIDC-IDRI	Radiomic	In all, there are 1018 low-dose lung computing tomography scans from 1010 lung patients in LIDC-IDRI.
[22]	WBCD	Clinical	In WBCD, to determine features, a digital image of a fine needle aspirate (FNA) of a breast mass is employed.
[23]	MIAS	Radiomic	MIAS contains original “Portable Gray Map” (PGM) format photos (322 in total (161 pairs)) captured at a 50-micron resolution as well as related ground-truth information.

**Table 3:** The summary and accuracy of cancer diagnosis using ANNs.

Type of Cancer	References	Key Contribution	Performance Metrics
Breast	[27]	Depending on the strength of the connection, it acts and matches, producing the match as necessary.	Accuracy = 95%
	[28]	In an ANN, a single neuron receives input from other neurons, multiplies it by the appropriate weight Wij, and then uses an activation function to create a weighted output f. (Xj).	AUC value, Accuracy, Sn value, Sp value
	[29]	Pre-processing was performed to increase accuracy by 2%.	More than 98%
Ovarian	[30]	Classification of an ovarian tumor.	Accuracy = 80% (dermoid cyst) Accuracy = 70% (Follicular cysts)
Pancreatic	[31]	Identification of pancreatic cancer.	Sensitivity = 87.3% Specificity = 80.8% AUC = 0.86
Lung	[32]	To reduce false-positive nodules, previously extracted characteristics were used.	73% sensitivity to detection
Liver	[33]	Hepatitis data analysis.	Accuracy = 86.3

**Table 4:** The summary and accuracy of cancer diagnosis using Naïve Bayes.

Type of Cancer	References	Key Contribution	Performance Metrics
Lung	[36]	Prediction of false and true positives	89.64% Accuracy
Breast	[37]	Data were collected with the help of doctors	98.2% accuracy
Liver	[38]	Liver disease prediction model based on the CatBoost algorithm	Precision 0.67 Recall 0.66 F1-score 0.63

**Table 5:** The summary and accuracy of cancer diagnosis using a decision tree.

Type of Cancer	References	Key Contribution	Performance Metrics
Ovarian	[41]	Classify types of ovarian tumors and cancer.	Accuracy = 89.52% (Using Median)
	[42]	Comparison of the performances of different machine learning models.	Accuracy = 99.1% Sensitivity = 100% Specificity = 97.9% (Using Simple Tree)
Lung	[43]	Contrast with other valued classification techniques such as SVM, KNN, MLP, and Logistic Regression.	Accuracy = 90.688%
Breast	[29]	Compared to ANN and linear regression, this method generated the best results.	Accuracy = 93.6%

**Table 6:** The summary and accuracy of cancer diagnosis using K-Nearest Neighbor.

Type of Cancer	References	Key Contribution	Performance Metrics		
Brain	[11]	Tumor classification into different classes: normal region and abnormal region.		Statistical Features	Gabor Wavelet Features
			Linear Kernel	94.8 ± 0.3%	91.7 ± 0.4%
			RBF Kernel	96.1 ± 0.2%	95.3 ± 0.6%
Brain	[108]	Novel and effective method for brain tumor segmentation and identification in MRI images.	Accuracy = 88.71%		
Breast	[29]	K ranged from 1 to 15 and best was equaled when k = 1.	Accuracy = 98.25%(KNN) Accuracy = 98.83% (fuzzy KNN)		
	[37]	Data were collected with the help of doctors.	Accuracy = 98.8%		
Ovarian	[41]	This method was used to classify types of ovarian tumors and cancer	Accuracy = 86.666% (Using Median)		
	[42]	The accuracy and false-positive rates of various machine learning models.	Accuracy = 93.5% Sensitivity = 93.5% Specificity = 93.5% (Using Weighted KNN)		
	[46]	Assessment of classification-model performance using data and selected features.	Balanced Accuracy = 84% Sensitivity = 90% Specificity = 77% F1 Measure = 92%		
		Assessment of classification-model performance using random features.	Balanced Accuracy = 71% Sensitivity = 96% Specificity = 46% F1 Measure = 91%		
Oral	[43]	Predict pathologic staging and TNM.	Accuracy = 85%		
Prostate	[47]	Classifies positive and negative samples.	Accuracy = 78.75		
Liver	[38]	Liver disease prediction model based on the CatBoost algorithm.	Precision 0.72 Recall 0.71 F1-score 0.70		

**Table 7:** The summary and accuracy of cancer diagnosis using Random Forest.

Type of Cancer	References	Key Contribution	Performance Metrics
Brain	[11]	Used for glioma classification into benign and malignant types.	AUC = 0.91
Ovarian	[30]	Early detection and prediction for Poly Cystic Ovarian Syndrome.	Accuracy = 89.02%
	[41]	Used to classify types of ovarian tumors and cancer.	Accuracy = 90.476% (Using Median)
	[46]	Assessment of classification-model performance using data and selected features.	Balanced Accuracy = 89% Sensitivity = 96% Specificity = 83% F1 Measure = 96%
		Assessment of classification-model performance using random features.	Balanced Accuracy = 70% Sensitivity = 100% Specificity = 41% F1 Measure = 92%
	[49]	Ovarian cancer classification using pathophysiological complications.	Accuracy = 72% Precision = 72% Recall = 85% F1 Score = 78%
Oral	[43]	Contrast with other valued classification techniques such as SVM, KNN, MLP, and Logistic Regression.	91% accuracy

**Table 8:** The summary and accuracy of cancer diagnosis using Support Vector Machine.

Type of Cancer	References	Key Contribution	Performance Metrics
Breast	[28]	DDSM dataset classification as normal or abnormal.	AUC = 94.4%
	[51]	SMV used on a DDSM dataset to classify into benign or malignant.	Accuracy = 93.1% Area under ROC curve = 0.7
	[37]	Gene expression and DNA methylation data were filtered to obtain common genes to determine the presence of tumors.	Accuracy = 99.68% on spark Accuracy = 98.03% on Weka tool
	[37]	Recursive feature extraction was used to evaluate SVM.	Accuracies of 99% on linear kernel, 84% on sigmoid and 98% on RBF respectively
Lung	[52]	SVM was helpful in prediction based on continuous and categorical data.	The overall accuracy was 100%
	[36]	SVM was used to propose graph-based image representation with deep point cloud models.	Average of 95% accuracy
	[32]	SVM’s data-driven methodology and viability without a fictitious system that generates an accurate classification of cancer.	97%-identification 87%-prediction
Ovarian	[49]	Classification of ovarian cancer based on pathophysiological complications.	Accuracy = 71% Precision = 71% F1 Score = 78% Recall = 86%
	[41]	SVM was used to classify different kinds of ovarian tumors and cancer.	Accuracy = 83.809% (SVC RBF)
	[42]	Performance evaluation of various machine learning models.	Accuracy = 94.4% Specificity = 89.7% Sensitivity = 94.1% (Using Quadratic SVM)
	[46]	Assessment of classification-model performance using data and selected features.	Balanced Accuracy = 85% Sensitivity = 96% Specificity = 74% F1 Measure = 94%
		Assessment of classification-model performance using random features.	Balanced Accuracy = 81% Sensitivity = 97% Specificity = 66% F1 Measure = 94%
Oral	[43]	Using a microarray analysis and expression profile on the patient, an SVM classifier was used to identify OSCC tumors.	Accuracy = 88%
	[53]	The blue automatic technique component of the experimental outcome was successful.	Accuracy = 98.01%
Liver	[54]	SVM was used to predicting tumor state in liver-cancer patients.	SD = 0.151 AMAUC = 0.897 SD = 0.143 AMACC = 0.895
Liver	[54]	SVM was tasked with predicting the states of tumors in colon-cancer patients	SD = 0.153 AMAUC = 0.897 SD = 0.159 AMACC = 0.890
Liver	[55]	Based on liver MR scans, a classification approach for diagnosing early-stage liver cancer was used.	Accuracy = 80%

**Table 9:** The summary and accuracy of cancer diagnosis using Ensemble Models.

Type of Cancer	References	Key Contribution	Performance Metrics
Ovarian	[42]	Performance evaluation of various machine learning models.	Accuracy = 93% Specificity = 89.9% Sensitivity = 95.7% (Using Subspace Discriminant)
Prostate	[58]	Substantially less expensive and more effective for predicting urinary toxicity.	Mean AUC = 0.63 ± 0.05

**Table 10:** Illustrates the summary and accuracy of cancer diagnosis using Logistic Regression.

Type of Cancer	References	Key Contribution	Performance Metrics
Prostate	[47]	It provides a methodological framework for modelling event likelihood.	Accuracy = 91.99%
Liver	[38]	Liver disease prediction model based on the CatBoost algorithm	Precision = 0.65 Recall = 0.63 F1-score = 0.62

**Table 11:** The summary and accuracy of cancer diagnosis using hybrid models.

Type of Cancer	References	Models Used	Key Contribution	Performance Metrics
Brain	[11]	SVM and KNN	Hybrid models is used to make classifications during the process of acquiring HSI from patients for real-time image analysis, SVM is used to obtain a supervised classification map, and KNN is used for finding the spatial–spectral classification map.	Accuracy = 97.5%
Breast	[28]	KNN and SVM	WBDC dataset,	Accuracy = 97.38%
	[62]	Rough Set and SVM	RS is used to determine the best features, and SVM increased its accuracy.	Accuracy = 98.53%

**Table 12:** The summary and accuracy of cancer diagnosis using recurrent neural networks.

Type of Cancer	References	Key Contribution	Performance Metrics
Lung	[32]	RNN is used to examine and study medical imaging including computed tomography (CT), magnetic resonance imaging (MRI), and X-ray images.	Accuracy = 98.08%

**Table 13:** The summary and accuracy of cancer diagnosis using a deep autoencoder.

Type of Cancer	References	Key Contribution	Performance Metrics
Ovarian	[64]	The model consists of 3 convolutional layers, which include a max-pooling and up-sampling layer. Pooling size is 9, and operator of convolution is set as 1. After feature extraction, ELM and SVM generated the best results.	Average using ELM = 95.6811
Breast and Lung	[67]	An Eight-category classification approach was implemented to assess (a) whether a cell is healthy or malignant and (b) the type of cell (and consequently cancer type and state) it is associated with.	Accuracy = 87.5% Sensitivity = 80% MCC = 0.775
			Sensitivity = 94.73% Accuracy = 91.3% MCC = 0.697

**Table 14:** The summary and accuracy of cancer diagnosis using long short-term memory.

Type of Cancer	References	Key Contribution	Performance Metrics
Multiple Types mainly thyroid, lung and breast	[71]	There was just one hidden layer composed of 100 LSTM units. The sigmoid function was set as the default in the output layer.	AUC = 0.74
Prostate	[72]	LSTM outperformed conventional cox-proportional hazards	AUC = 0.936

**Table 15:** The summary and accuracy of cancer diagnosis using deep neural networks.

Type of Cancer	References	Key Contribution	Performance Metrics
Lung	[74]	DNNs offer a way to validate the robustness of OPF in the classification problem.	Accuracy = 98.08%
Oral	[75]	DNN uses the Gravitational Search Optimized Echo State Neural Networks to analyze oral X-ray pictures and predict oral cancer.	Accuracy = 99.2%
Prostate	[76]	DNN is used to determine the survival times of people with prostate cancer.	Accuracy = 85.64%
Ovarian	[30]	DNN is used to detect cysts in females.	Accuracy = 92.11%
Liver	[77]	DNN is used to examine haemangioma, hepatocellular, and metastatic carcinoma tumors.	Accuracy = 99.39%

**Table 16:** The summary and accuracy of cancer diagnosis using deep belief networks.

Type of Cancer	References	Key Contribution	Performance Metrics
Lung	[80]	DBNs are used to extract features for lung cancer detection.	Accuracy = 81.19%
Breast	[80]	DBs are used to extract features for breast cancer detection.	KPI Accuracy = 99.68%

**Table 17:** The summary and accuracy of cancer diagnosis using deep convolutional neural networks.

Type of Cancer	References	Key Contribution	Performance Metrics
Lung	[84]	DCNNs support medical imaging techniques that tend to use structured databases.	All slicers of 75 patient folders of a LIDC-IDRI dataset were extracted and presented with lung reports and stored in .png file formats at 8-bit resolution. A total of 282 images with 282 masks were obtained.
	[36]	DCNNs addressed the issue of overfitting that developed throughout the training process because of a dataset imbalance.	Average AUC = 85.0%
	[74]	DCNNs are used to classify NSCLC lymph node metastases	Accuracy = 94.73%
	[32]	A tumor is located using an attention-guided DCNN (AG-CNN).	Accuracy = 74.43 ± 6.01
	[85]	DCNNs have significantly surpassed the prior generation of Radiomic/texture analysis techniques.	AUC of low 90 s
	[107]	Pulmonary nodules are classified as malignant or benign in lung cancer.	Accuracy = 92.4% (Nodule Classification)
Oral	[86]	DCNNs have the ability to create their own representations, which are required for pattern recognition.	
	[87]	The strategy used by the DCNN involved looking for images, training, data, quotations, and classification.	Accuracy = 97.21%
	[53]	The combination of WLI and AFI provide the most details on the type of lesion and the extent of the damaged area once problematic spots have been identified.	AUC = 90.8%
Prostate	[88]	DCNNs radically improve the detection and treatment of prostate cancer.	Accuracy = 95%
Ovarian	[89]	DCNNs are used to identify ovarian disease using cytological images	Accuracy = 78.2%

**Table 18:** The summary and accuracy of cancer diagnosis using Deep Boltzmann Machines.

Type of Cancer	References	Key Contribution	Performance Metrics
Breast	[92]	ClassRBM was trained with a learning rate of between 0.01 and 0.1, a momentum rate of 0.5, and 100,000 iterations.	Accuracy = 0.738 ± 0.015 (highest using classRBM + DropOut)
Oral	[93]	To train the Deep Boltzmann Machine network for class construction, 850 nm-wavelength cancer cell hyperspectral pictures were utilized.	Accuracy% = 95.5 Sensitivity% = 93.5 Specificity% = 94.94 False positive (FP) = 2.14

**Table 19:** The summary and accuracy of cancer diagnosis using hybrid deep learning models.

Type of Cancer	References	Models Used	Key Contribution	Performance Metrics
Breast	[94]	YOLO, CNN, ResNet-50, and InceptionResNet-V2.	Two datasets, DDSM and INbreast, are used.	CNN 88.7% ResNet-50 92.5% InceptionResNet-V2 95.3%
Breast	[95]	Extreme learning machine (ANN).	Breast cancer detection.	Accuracy = 96.92%
Prostate	[96]	Radial basis function neural network (RBF).	Enhanced classification accuracy.	Accuracy = 94.1%l

**Table 20:** Smart phone applications in cancer diagnosis and therapy.

References	Smart Phone Application-Name	Mobile Operating System	Availability	App Description and Features	Users	Utility
[110]	SkinVision	iOS 13.0 or Above Android 7.0 or Above	Partially Free	Skin tracking and skin spot analysis.	Patients	Assessment
[111]	Breast Check Now	iOS 10.0 or Above Android 4.4 or Above	Free	Breast self-exam instructions.	Women	Awareness
[112]	Ada Health	iOS 13.0 or Above Android 5.0 or Above	Free	Symptom assessment tool and health library.	Patients	Assessment
[113]	Cancer.Net Mobile	iOS 10.0 or Above Android 5.0 or Above	Free	Cancer-related resource with a tool for tracking symptoms, appointments, and medications.	Patients and Physicians	Information
[114]	ASCO Guidelines	iOS 12.0 or Above Android 5.0 or Above	Free	Tools for risk assessment, staging, and treatment-related decision-making based on the latest clinical evidence.	Physician	Guidance

## Data Availability

Not applicable.

## Appendix A

**Table A1:** A list of the acronyms used in this article along with their definitions.

**Acronym**	**Definition**
AI	Artificial Intelligence
ANN	Artificial Neural Network
BP	Back-Propagation
CNN	convolutional neural network
SVM	Support Vector Machine
RNN	recurrent neural network
LR	Logistic Regression
LSTM	Long short-term memory
